# Protein Tyrosine Phosphatases in Cancer: From Oncogenic Drivers to Therapeutic Targets

**DOI:** 10.3390/cells15191767

**Published:** 2026-09-29

**Authors:** Sravan Perla, Jonathan Pascale, Matthew Liu, Ashish Kumar, Sivaraman Kuppuswamy, Charanraj Goud Alladi, Vishnu Amaram Samara

**Affiliations:** 1 Department of Pharmacology, Yale University School of Medicine, New Haven, CT 06510, USAashish.kumar@yale.edu (A.K.); 2 Department of Biochemistry and Molecular Biophysics, Columbia University Irving Medical Center, New York, NY 10032, USA; mtl2175@cumc.columbia.edu; 3 Centre for Molecular and Nanomedical Sciences, International Research Centre, Sathyabama Institute of Science and Technology (Deemed to Be University), Chennai 600119, Tamil Nadu, India; shivabiochem45@gmail.com; 4 Institute of Molecular and Translational Medicine, Faculty of Medicine and Dentistry, Palacký University and University Hospital Olomouc, 779 00 Olomouc, Czech Republic; charan.raj002@gmail.com; 5 Department of Pathology, University of Chicago, Chicago, IL 60637, USA

**Keywords:** protein phosphatases, Phosphorylation, cancer, tumor suppressor, signal transduction, SHP2, PTP1B, PTEN, small-molecule inhibitors, targeted therapy

## Abstract

Protein tyrosyl phosphorylation is a dynamic reversible regulatory process that is essential for virtually all aspects of cellular function. The dysregulation of protein tyrosyl phosphorylation is one of the main causes of human diseases such as cancer, diabetes, autoimmune disorders, and neurological diseases. Protein tyrosine kinases (PTKs) have paved the way towards defining the significance of protein tyrosyl phosphorylation in human health and disease. However, homeostatic control of protein tyrosyl phosphorylation is also regulated by protein tyrosine phosphatases (PTPs). The actions of PTPs are diverse functioning to both positively and negatively regulate signaling pathways through specific substrate dephosphorylation. PTKs are now matured targets with numerous drugs that have been developed for the treatment of human diseases such as cancer and immunological disorders. In contrast, PTPs are relatively in their infancy as compared with their PTK counterparts with regard to drug development for the treatment of human diseases. However, mounting evidence supports the view that PTPs are not only valid targets but are indeed targetable. This review focuses primarily on the roles of PTPs as oncogenic drivers and therapeutic targets in cancer, while briefly noting their broader relevance to other diseases in which tyrosyl phosphorylation is dysregulated. This review will highlight recent developments in the PTP field, with an emphasis on cancer, which provide support for the emergence of PTPs as therapeutic targets, and will also briefly touch upon their relevance to related conditions such as obesity, type II diabetes and rare diseases.

## 1. Introduction

Signal transduction is an intricate process that transmits extracellular signals through a series of events involving protein tyrosine phosphorylation and dephosphorylation to produce a broad spectrum of signal responses. Protein tyrosine kinases (PTKs) transfer phosphate groups from ATP to target tyrosine residues in proteins, thereby activating signal transduction pathways linked to cell growth, proliferation, differentiation, metabolism, and cell death. Protein tyrosine phosphatases (PTPs) are a group of enzymes that mediate the dephosphorylation of proteins on tyrosine residues. PTPs play an important role in the regulation of cell growth, proliferation, and differentiation [1]. A total of 95 PTKs are counteracted by 107 PTPs to maintain the net balance of cellular tyrosyl phosphorylation [2]; this structural diversity of 107 PTPs encompasses not only classical catalytically active phosphatases but also catalytically inactive pseudophosphatases and the dual-specificity phosphatases (DUSPs) discussed below, reflecting the broader functional range of the PTP superfamily beyond simple phosphotyrosine hydrolysis. The PTP superfamily is characterized by a consensus signature motif HC(X)5R, which designates the active site of these enzymes [3]. PTPs can be grouped into two classes. The first class is characterized by cysteine-based phosphotyrosine selectivity, and the second class constitutes the dual-specificity phosphatases (DUSPs) [4]. The traditional PTP genes can be further classified into non-transmembrane PTPs and transmembrane receptor PTPs (RPTPs) [5].

Mutations in both the PTKs and PTPs are documented to be linked with various human diseases [6,7]. The significance of PTKs in the context of cancer has been thoroughly investigated, but studies on PTPs have lagged behind PTKs since these proteins were assumed to function as housekeeping proteins [8,9]. However, it is now abundantly clear that PTPs are critical for the maintenance of protein tyrosyl phosphorylation levels and work in concert with the PTKs [4]. The success of PTKs as drug targets for the treatment of human diseases such as cancer and immunological disorders has provided a conceptual platform from which to investigate whether PTPs can be similarly targeted therapeutically. Although the PTP family is extensive, much of the focus regarding the development of therapeutics for PTPs against diseases such as cancer, immunological disorders and metabolic disease has largely focused on a handful of PTPs. These PTPs include SHP-2, PTP-1B, TC-PTP, PRLs, STEP and more recently the DUSPs, in particular MKP-5. As such, these PTPs represent the forefront of PTP therapeutic development and serve as excellent representations of the advancements and trajectory of PTP research.

## 2. Protein Phosphatases

Protein phosphatases are a group of enzymes that remove phosphate groups from phosphorylated amino acid residues, counterbalancing the action of kinases and playing essential roles in regulating cellular signaling [4]. Based on substrate specificity and structural characteristics, protein phosphatases are broadly classified into three major families: serine/threonine protein phosphatases (PSPs), protein tyrosine phosphatases (PTPs), and dual-specificity phosphatases (DUSPs). PSPs, and PP2A in particular, are briefly introduced here because they physically and functionally intersect with PTP-regulated networks in cancer—for example, by dephosphorylating shared nodes such as AKT and ERK—and because PP2A inhibition is itself being explored as an anticancer strategy in parallel with PTP-directed approaches [10,11]; this family is therefore included for comparative context rather than as a primary focus of this review, which centers on PTPs.

### 2.1. Serine/Threonine Protein Phosphatases (PSPs)

Serine/threonine protein phosphatases (PSPs) primarily dephosphorylate phosphoserine and phosphothreonine residues [12]. This family includes the phosphoprotein phosphatase (PPP) family (e.g., PP1, PP2A, PP2B/calcineurin, PP4, PP5, PP6) and the protein phosphatase, Mg^2+^/Mn^2+^-dependent (PPM) family (e.g., PP2C, PPM1D) [12]. In cancer, PSPs can act as either tumor suppressors or promoters, depending on context. For example, PP2A is well known for its tumor-suppressive role by negatively regulating key oncogenic pathways, such as the AKT and ERK pathways. However, paradoxically, inhibiting PP2A has also shown potential as a therapeutic strategy in certain cancers [13,14]. In addition, the dysregulation of some PSPs, such as overexpression of PPM1D, has been implicated in tumorigenesis and therapy resistance [15,16].

### 2.2. Protein Tyrosine Phosphatases (PTPs)

Protein tyrosine phosphatases (PTPs) selectively dephosphorylate phosphotyrosine residues. This group encompasses classical receptor-type (e.g., PTPRA, PTPRF, PTPRB) and non-receptor-type PTPs (e.g., PTP1B, SHP1/PTPN6, SHP2/PTPN11) (Figure 1) [17]. PTPs have diverse roles in cancer: while some, such as PTEN, function as well-established tumor suppressors through PI3K/AKT pathway inhibition, others like SHP2 (encoded by PTPN11) can promote tumor growth and are emerging therapeutic targets in RAS-driven cancers [18,19].

### 2.3. Dual-Specificity Phosphatases (DUSPs)

Dual-specificity phosphatases (DUSPs) can dephosphorylate both phosphoserine/threonine and phosphotyrosine residues. This family includes DUSP1–DUSP28, among others, many of which are MAPK phosphatases that regulate ERK, JNK, and p38 pathways (Figure 2) [20,21]. Dysregulated expression of DUSPs contributes to cancer progression by affecting cell proliferation, survival, and drug resistance [21]. For example, DUSP6 specifically inactivates ERK1/2 and can act as a tumor suppressor in certain contexts. However, in some cases, high levels of DUSP6 are associated with increased cancer cell growth [22,23].

Together, these Phosphatase families tightly regulate signaling cascades involved in proliferation, apoptosis and DNA damage responses. In cancer, their roles are complex and often context-dependent, making them attractive as targets for therapeutic intervention.

## 3. Protein Tyrosine Phosphatases in Cancer

SHP2: Src homology region 2 (SH2)-containing protein tyrosine phosphatase 2 (SHP2) is a ubiquitously expressed non-receptor PTP that is encoded by the PTPN11 gene [1]. SHP2 transduces signals from several growth factor, cytokine, and extracellular matrix receptors that are mitogenic, pro-survival, pro-cell-fate, and/or promigratory [1,24]. SHP2 has two adjacent SH2 domains (N-SH2 and C-SH2) at its N-terminus, and a PTP catalytic domain, followed by two tyrosine phosphorylation sites (Y542 and Y580) and a proline-rich region at its C-terminus [25]. In the normal state SHP2 is in a closed conformation in which the catalytic domain is blocked by the N-SH2 domain [25]. When growth factors or cytokines stimulate the cell, phosphorylated tyrosine residues on proteins like receptor tyrosine kinases (RTKs), cytokine receptors, and scaffolding adaptors bind to the N-SH2 domain [26]. This binding disrupts autoinhibition and activates the enzyme [26].

Mutations in the PTPN11 gene lead to Noonan Syndrome and Noonan Syndrome with Multiple Lentigines, and since SHP2 is involved in many signaling pathways, it is implicated in a variety of cancer types [27,28,29]. In acute myeloid leukemia (AML), SHP2 functions as an oncogenic mediator by binding to PD-1 expressed on leukemia stem/progenitor cells, initiating downstream signaling that promotes leukemic proliferation and survival, bypassing immune control and promoting proliferation [30]. In clear cell renal cell carcinoma (ccRCC), SHP2 acts downstream of RGC32 to activate NF-κB/EGFR signaling, enhancing proliferation, invasion, and epithelial–mesenchymal transition (EMT) [31]. In cervical cancer, SHP2 in tumor-associated macrophages (TAMs) participates in the PD-1/IRE1α/SHP2/HIF1α axis to promote angiogenesis and cell migration [32]. Inhibition of this pathway with PD-1 monoclonal antibodies increases SHP2 levels yet suppresses HIF1α-driven neovascularization, demonstrating SHP2’s involvement in tumor vascular biology and metastasis regulation [32].

In BRAF(V600E) mutant colorectal cancer, SHP2 activation is necessary for the oncogenic MUC1-C protein to drive proliferation and BRAF inhibitor resistance [33], while in other colorectal cancers, high SHP2 expression correlates with reduced metastasis and improved survival, illustrating SHP2’s context-dependent role in cancer progression [34,35]. Moreover, SHP2 also sustains ERK/MAPK signaling as an oncogenic amplifier in esophageal squamous cell carcinoma, but in myeloid cells, SHP2 downregulates STAT3/STAT6 signaling, and deficiency in SHP2 has been shown to promote esophageal carcinoma [36,37].

Additional oncogenic roles include folic-acid-driven proliferation via the caveolin-1-SHP2-p-cSrcY416 pathway and drug resistance in breast cancer, sustained FGR and KRAS signaling in non-small cell lung cancer (NSCLC) to drive drug resistance and developing anoikis resistance and EMT through TGF-β1/SHP2/JAK2/STAT3 and SHP2/Grb2 signaling pathways [38,39,40,41,42]. Additionally, cancer cell-derived exosomal circUSP7 upregulates the expression of SHP2, which causes immunosuppression by promoting CD8+ T cell dysfunction [41].

SHP2 also drives hypoxia-induced tumor aggressiveness in oral cancers and suppresses interferon-alpha anti-proliferative signaling in hepatocellular carcinoma and renal cancer [43,44,45]. In thyroid cancer, PD-1 blockade suppresses an intrinsic SHP2/Ras/MAPK program, delaying tumor growth, while in anaplastic thyroid carcinoma, SHP2 inhibition enhances immunogenicity and dendritic cell-mediated phagocytosis [46,47].

More recently, SHP2 inhibition has also gained attention as a rational combination strategy in RAS- and RTK-driven malignancies. Because SHP2 functions downstream of multiple RTKs and facilitates RAS/MAPK pathway activation, SHP2 inhibitors are being evaluated with RAS-pathway inhibitors and RTK-directed therapies to overcome adaptive pathway reactivation and therapeutic resistance. In KRAS-mutant lung cancer, clinical studies have examined SHP2 inhibitors in combination with KRAS G12C inhibitors, including sotorasib plus RMC-4630 in KRAS G12C-mutant NSCLC and other advanced solid tumors, as well as broader studies combining SHP2 inhibitors with ERK, EGFR, HER2, VEGF, PI3K, or PD-1 pathway inhibitors [48]. Similarly, the SHP2 inhibitor batoprotafib (TNO155) has been evaluated alone and in combination with the EGFR inhibitor nazartinib (EGF816) in advanced solid tumors, supporting the concept that SHP2 blockade may suppress RTK-mediated signaling dependencies [48]. In breast cancer, SHP2 inhibition has also been proposed as a way to overcome RTK-mediated resistance, including resistance to PI3K inhibition in luminal and triple-negative breast cancer models, further supporting SHP2 as a therapeutic node in both lung and breast cancer signaling networks [49].

PTP1B: Protein tyrosine phosphatase 1B (PTP1B), which is encoded by the PTPN1 gene, is a highly studied phosphatase. It has been observed to function as either a tumor suppressor or promoter, depending on the specific cellular environment. Structurally and mechanistically related to TC-PTP, PTP1B plays pivotal roles in diverse oncogenic signaling pathways, including PI3K/AKT, Src, and Hippo-YAP [50].

In hepatocellular carcinoma (HCC), PTP1B overexpression enhances tumor growth, proliferation, migration, invasion, and angiogenesis by sustaining PI3K/AKT activation and suppressing AMPK activity [51]. In breast cancer, the PTP1B/PI3K/AKT pathway induces proliferation and suppresses apoptosis, and these effects can be reversed using derivatives of oleanolic acid glycosides, such as OA-Br-1 [52]. Consistently, PTP1B expression correlates with immune infiltration and checkpoint gene expression, further linking PTP1B activity to tumor immune microenvironment modulation [50]. PTP1B also promotes tumor progression in glioblastoma by activating Cdk3 through dephosphorylation, leading to Rb phosphorylation, E2F-dependent transcription, and G1/S transition, thereby accelerating proliferation [53]. Another study supported this by showing that miR-34c-mediated inhibition of PTP1B suppresses glioma cell proliferation [54]. In melanoma, PTP1B promotes tumor growth and metastasis by dephosphorylating Src at Tyr527, maintaining its active conformation and driving downstream pro-metastatic signaling [55]. Similarly, PTP1B has been shown to have a role in tumor immune evasion in colorectal cancer [56]. On the other hand, PTP1B can also exert tumor-suppressive effects in some cancers. In cholangiocarcinoma, PTP1B interacts with the Hippo pathway effector Yes-associated protein (YAP), which is a driver for oncogenesis; PTP1B inhibits YAP activity, decreasing cholangiocarcinoma cellular proliferation [57].

TC-PTP/PTPN2: The PTPN2 gene codes for human T-cell protein tyrosine phosphatase (TC-PTP) belonging to the family of classical non-receptor PTPs [58]. TC-PTP contains a conserved catalytic domain and a variable length C-terminus. PTPN2 is closely related to PTP1B, possessing similar enzymatic activity and around 72% sequence identity in their catalytic domains [59,60]. Because of alternative splicing at its carboxyl terminus, PTPN2 encodes two distinct isoforms in humans and rodents: a 48.5 kDa TC-PTP (TC48) and 45 kDa TC-PTP (TC45). Key variations that distinguish the two isoforms are found at their C-terminus. In TC45, the inclusion of a nuclear localization signal (NLS) allows it to act within the nucleus and cytoplasm, gaining access to a wide variety of substrates. TC48 also has an NLS but uniquely has a hydrophobic C-terminus element, which leads to it being localized to the endoplasmic reticulum [59,60].

The role of TC-PTP in cancer is quite complex, with demonstrated potential to act not only as a tumor suppressor but also as a driver of cancer progression [61,62]. TC-PTP has been shown to act as a suppressor of tumor development in that a loss of TC-PTP was found to promote the development of breast, liver, and skin cancers, often through influencing STAT-3 signaling [63]. In fact, several components of the JAK-STAT pathway are TC-PTP substrates and have gained increased attention as prospective targets in cancer immunotherapeutic approaches [64]. In other studies, TC-PTP has shown itself to be a promoter of cancer progression, such as within KRAS-dependent cancer cell lines where TC-PTP promoted membrane localization of KRAS, and a loss of TC-PTP corresponded with marked attenuation of cancer cell proliferation and enhanced apoptosis [65]. Despite the very clear and mounting evidence to suggest that TC-PTP plays a critical role in cancer suppression and progression, there remains no commercially available agents targeting TC-PTP, though work is being done to fill this gap. Notably, researchers are exploring the use of proteolysis-targeting chimera (PROTAC) technology to overcome intrinsic challenges associated with phosphatase-targeting drug design and have taken the first steps towards the development of immunotherapeutic agents targeting the phosphatase [58].

Several tumor-suppressor PTPs have been identified whose loss has been shown to be associated with abnormal growth of tumors, metastasis in in vitro and in vivo models (Table 1). Recent studies have shown that PTPs can also act as oncogenes to induce tumor growth. A few human cancers have abnormally high expression of these oncogenic PTPs, which promotes tumor development, growth, and metastasis and, ultimately, lowers survival. In case of breast cancer, leukemia and gliomas, SHP2 acts as an oncogene and dysregulates the EGFR/Ras/MAPK pathway [66,67,68,69,70,71,72]. PTP1B acts as oncogene in ovarian, gastric, prostate and breast cancer and dysregulates the Src/Ras&PI3/Akt pathway [73,74,75,76,77,78,79,80]. PTP4A3 acts as oncogene in breast, gastric and colon cancers and dysregulates the PDGF, integrin and EphA signaling pathway [81,82,83,84] (Figure 3).

Table 1: Cancer types and PTPs are listed as reported in the cited primary studies; entries appear more than once, singly, or in groups because individual PTPs have been characterized as tumor suppressors across multiple independent cancer types and studies, and each row reflects a distinct cancer–PTP pathway association as described in its corresponding reference(s), rather than a fixed classification scheme. Abbreviations: AKT, protein kinase B; BCR:ABL1, breakpoint cluster region–Abelson fusion oncoprotein; EGFR, epidermal growth factor receptor; FGFR, fibroblast growth factor receptor; FOXM1, forkhead box M1; HER2, human epidermal growth factor receptor 2; MAPK, mitogen-activated protein kinase; mTOR, mechanistic target of rapamycin; NSCLC, non-small cell lung cancer; P53, tumor protein p53; PI3K, phosphoinositide 3-kinase.

The development of PTP inhibitors is still difficult, even though oncogenic PTPs are appealing molecules for the creation of tailored treatments. Because of the great degree of conservation in the active site of both tumor suppressor and oncogenic PTP subtypes, selectivity presents a significant obstacle in the development of PTP inhibitors. Thus, the inhibitors that target the active sites of oncogenic PTPs also inhibit tumor-suppressor PTPs in healthy tissue and potentially result in unfavorable side effects. However, these negative side effects could be avoided by generating selective PTP inhibitors that bind to regions outside of the PTP active site [100].

## 4. Future of PTP Inhibitors for Cancer Therapy

Recent studies have demonstrated that oncogenic PTPs are appealing candidates for the development of targeted therapeutics for many cancer types (Figure 4). Safe and effective PTP inhibitors remain scarce for clinical use, as the development of selective PTP inhibitors has proven difficult. Phase I clinical trial results with the SHP2 inhibitor sodium stibogluconate (SSG; Lenocta™, supplied by VioQuest Pharmaceuticals, Inc., Basking Ridge, NJ, USA) demonstrated that patients with stage IV melanoma and advanced solid tumors could tolerate doses of 1200 and 900 mg/m^2^, respectively; as a Phase I study, this trial was designed to establish maximum tolerated dose and safety, not efficacy, and its findings should not be read as evidence of clinical benefit [100,101,102]. Comparably, early-phase results from the PTP1B inhibitor MSI-1436C (Trodusquemine; DepYmed, Inc., Farmingdale, NY, USA) phase I clinical trial reported the drug’s safety and tolerability in the treatment of metastatic breast cancer, will be instrumental to the translation of this drug to the clinical setting. To successfully generate highly specific PTP inhibitors for cancer therapy, more research is needed to unravel specific essential locations situated outside of the active sites, even though various other phosphatases have also been demonstrated to be interesting targets.

Targeting PTPN2 can improve the efficacy of human chimeric antigen receptor (CAR) T cells against solid tumors. CRISPR-Cas9-mediated deletion of PTPN2, or pharmacologic inhibition using the PTPN2/PTP1B inhibitor ABBV-CLS-484, enhanced CAR and cytokine signaling in Lewis Y-specific CAR T cells [103]. PTPN2 targeting increased antigen-induced CAR T cell expansion, activation, cytotoxicity, interferon-associated signaling, and tumor repression in human tumor xenograft and patient-derived xenograft models [103]. Importantly, PTPN2 deletion or inhibition also promoted the persistence of CD8+ CAR T cells with long-lived stem cell memory-like features, suggesting that PTPN2 inhibition may help address major limitations of CAR T therapy in solid tumors, including poor persistence and tumor-microenvironment-mediated exhaustion. PTPN22 has also emerged as another immune-restricted phosphatase target for cancer immunotherapy. Genetic loss or small-molecule inhibition of PTPN22 with L-1 augmented antitumor immunity by increasing CD8+ T cell activation and shifting tumor-associated macrophages toward MHC-II-expressing, M1-like phenotypes [102]. PTPN22 inhibition also enhanced the activity of anti-PD-1 therapy, and patients carrying the PTPN22 rs2476601 variant showed improved responses to checkpoint inhibition, supporting PTPN22 as a druggable systemic immunotherapy target [104].

Recent advances in the understanding of phosphatases in cancer have shown that these critical growth-regulatory molecules can, in principle, be pharmacologically targeted in a manner conceptually analogous to kinases, although this claim remains supported mainly by preclinical and early-phase clinical data rather than by validated, approved drugs, and should be interpreted with corresponding caution. However, targeting phosphatases is far more challenging than targeting kinases because many of them are constitutively active and are frequently controlled by binding partners or subunits. Previous studies have demonstrated that inhibition of oncogenic phosphatases can suppress the growth of human cancers. The central challenge is to develop safe and effective strategies to target these cancer-promoting phosphatases. Once this obstacle is cleared, a completely new class of cancer therapeutics will become available to address numerous serious cancers.

## 5. Dual-Specificity Phosphatases (DUSP) in Cancer

DUSP1: Dual-specificity Phosphatase 1 (DUSP1), also known as MKP-1, is a stress-inducible phosphatase that dephosphorylates JNK and p38 (with weaker activity toward ERK) to regulate MAPK signaling cascades [105]. In cancer, DUSP1 can act either as a tumor promoter or suppressor. In pancreatic ductal adenocarcinoma, DUSP1 promotes tumor growth by suppressing ferroptosis through inhibition of lipid peroxidation [104,105]. In high-grade serous ovarian carcinoma, DUSP1 overexpression drives paclitaxel resistance by promoting drug efflux via p38 MAPK signaling, and its inhibition blocks cell cycle progression by disrupting the AMPK/mTORC pathways [106,107]. In non-small cell lung cancer, DUSP1 is upregulated in osimertinib-resistant cells and mediates therapeutic resistance by inhibiting MAPK-induced apoptosis [108]. In cervical cancer, it suppresses JAK2/STAT3 signaling by downregulating oncogenic miR-21, limiting migration and invasion [109]. In hepatocellular carcinoma, it promotes autophagy via the AMPK–ULK1–Beclin-1 axis, leading to tumor suppression [110]. Additionally, in endometrial and prostate cancers, DUSP1 impairs EMT and promotes apoptosis by targeting EPHA2, ERK/JNK, and Snail, correlating with reduced metastatic potential [111,112].

DUSP2: Dual-specificity Phosphatase 2 (DUSP2) is a nuclear MAPK phosphatase that inactivates ERK1/2 (and to a lesser extent p38 and JNK) to modulate inflammatory and proliferative signaling in immune cells [113]. DUSP2 is predominantly regarded as a tumor suppressor across multiple cancers. In gastric cancer, DUSP2 suppresses ERK-driven EMT, invasion, and metastasis by restoring epithelial markers and repressing mesenchymal genes, with its expression negatively regulated by SKA3 [113]. In pancreatic ductal adenocarcinoma, DUSP2 inhibits AKT signaling and promotes apoptosis under hypoxia; conversely, AKT activation produces TRIM2, which degrades DUSP2, promoting survival and resistance [114]. Epigenetic mechanisms such as DNTTIP1–HDAC1-mediated transcriptional repression and circEIF3I–miR-361-3p post-transcriptional silencing suppress DUSP2 expression in nasopharyngeal and hepatocellular carcinoma, enhancing metastasis and tumor growth [115,116]. In prostate and bladder cancers, DUSP2 loss correlates with EMT, oxidative stress, immune evasion, and poor clinical outcomes, while its restoration inhibits tumor progression and sensitizes cells to therapy through modulation of MAPK, mTOR, and immune pathways [117,118,119].

DUSP6: Dual-specificity Phosphatase 6 (DUSP6), also known as MKP-3, is a cytosolic phosphatase that selectively dephosphorylates ERK1/2, acting as a negative feedback regulator of the RAS/ERK pathway [23]. In cancer, it can exhibit both tumor-suppressive and oncogenic functions. As a tumor suppressor, DUSP6 is frequently downregulated in RAS-driven pancreatic, lung, and liver cancers, where its loss results in sustained ERK signaling and enhanced proliferation and metastasis [120,121]. On the other hand, as a tumor promoter, DUSP6 overexpression in therapy-resistant cancers such as HER2-positive breast and cisplatin-resistant ovarian cancer dampens toxic ERK signaling, supports drug tolerance, and correlates with poor outcomes [122,123]. DUSP6 also contributes to metastasis and metabolic reprogramming by promoting Notch1 signaling in colorectal cancer, suppressing EMT in renal cell carcinoma, and modulating glycolysis via ERK-PKM2 in non-small cell lung cancer [3,124,125,126]. Furthermore, it facilitates DNA damage repair and therapy resistance by enhancing DNA-PKcs recruitment at double-strand breaks in glioblastoma and prolonging G2/M cell cycle arrest in esophageal squamous cell carcinoma [127,128].

DUSP16: Dual-specificity Phosphatase 16 (DUSP16), also known as MKP-7, dephosphorylates JNK and p38 MAPKs to regulate stress-induced apoptotic and inflammatory signaling cascades [129,130]. It contains an allosteric site centered around Tyr271 that is essential for MAPK binding and catalytic activity [131]. In nasopharyngeal, colorectal, gastric, breast, and head and neck tumors, DUSP16 is overexpressed and promotes chemoresistance by suppressing JNK/p38-mediated apoptosis and mitochondrial death signaling [132]. In hematologic cancers, DUSP16 maintains leukemic stemness and proliferation of CD34^+^ hematopoietic progenitors with expression epigenetically regulated by HDAC1/3 [133]. Alternatively, in osteosarcoma and endometrial cancer, DUSP16 acts as a tumor suppressor by inhibiting EMT and glycolytic reprogramming through regulation of the JNK-c-JUN axis [134,135]. Hypoxia-induced circular forms of DUSP16 (circDUSP16) also enhance glycolysis, drug resistance, and tumor growth by sponging tumor-suppressive miRNAs in gastric, colorectal, and breast cancers [136,137,138].

Viewed together, the four DUSPs discussed above illustrate a common structure–function logic that can inform therapeutic prioritization. Substrate specificity places DUSP1 and DUSP2 primarily upstream of JNK/p38 (with weaker ERK activity), whereas DUSP6 and DUSP16 act more selectively on ERK1/2 and JNK/p38, respectively; this distinction predicts which oncogenic pathway a given inhibitor would most directly affect. Cancer context is consistently bidirectional: each of these DUSPs can behave as a tumor suppressor in one setting (e.g., DUSP1 in pancreatic cancer, DUSP6 in RAS-driven pancreatic/lung/liver cancers, DUSP16 in osteosarcoma) and as a tumor promoter in another (e.g., DUSP1 in paclitaxel-resistant ovarian cancer, DUSP6 in HER2-positive breast cancer, DUSP16 in nasopharyngeal and hematologic malignancies), so therapeutic direction (activation versus inhibition) must be chosen on a tumor-type-specific basis rather than assumed from the gene family alone. Clinical maturity across this subclass remains low relative to SHP2 and PTP1B: to date, DUSP-directed strategies (e.g., the DUSP6 inhibitor BCI) are supported only by preclinical and cell-based data, with no compounds yet in early-phase clinical testing, underscoring that DUSPs are currently better regarded as promising but early-stage targets requiring further target validation before prioritization for drug development.

## 6. Therapeutic Prioritization, Toxicity, and Tumor-Specific Considerations

SHP2 and PTP1B currently represent the most clinically mature PTP targets and are reasonable near-term priorities for drug development, given their advancement into early-phase clinical trials (Section 3) and their well-characterized mechanistic roles in RAS/RTK-driven signaling; PP2A and the DUSPs, by contrast, remain earlier-stage targets supported primarily by preclinical data and should be prioritized for further target validation rather than immediate clinical translation. Tumor-specific applications also differ by target: SHP2 inhibition is most rationally applied in RAS-pathway- and RTK-driven malignancies such as KRAS G12C-mutant NSCLC and EGFR-driven cancers, where it can be combined with pathway-specific inhibitors to overcome adaptive resistance, whereas PTP1B-directed strategies are most supported in HER2-positive and triple-negative breast cancer and in hepatocellular carcinoma, where PTP1B sustains PI3K/AKT signaling. Toxicity is a central concern for both: because SHP2 and PTP1B also perform physiological signaling roles in normal tissue (e.g., SHP2 in hematopoietic and immune cell RAS/MAPK signaling [139], PTP1B in systemic insulin and leptin signaling [140,141]), active-site-directed inhibition risks on-target, off-tumor effects such as immune-related or metabolic adverse events, consistent with the toxicity liabilities generally associated with RAS/MAPK-pathway inhibitors in the clinic [142]. This risk reinforces the rationale, discussed above, for prioritizing allosteric or non-active-site inhibitor strategies that can achieve isoform- and context-selectivity.

## 7. Conclusions and Future Perspectives

### 7.1. Conclusions

Collectively, this review establishes PTPs as validated, high-value drug targets rather than passive “housekeeping” enzymes, though the field remains considerably less mature than that of kinases: 107 PTPs counterbalance 95 PTKs to maintain cellular phosphotyrosine homeostasis, yet PTP structural, functional, and inhibitor characterization lags far behind. A central theme of this review is that PTP function is highly context- and tissue-dependent: the same phosphatase, such as SHP2 or PTP1B, can act as an oncogene in one cancer type and a tumor suppressor in another, meaning PTP inhibition cannot be applied as a universal cancer strategy and rational drug development must instead account for tumor type and genetic driver context.

Among the PTP family, SHP2, PTP1B, and PP2A currently have the most mature mechanistic and translational data, positioning them as the most clinically advanced near-term candidates for drug development; even so, emerging evidence shows that the apparent role of a PTP can shift depending on genetic driver context, underscoring the need for caution when generalizing PTP function across tumor settings. The principal technical barrier to translating this biology into safe therapeutics remains selectivity: because the catalytic active sites of oncogenic and tumor-suppressor PTPs are highly conserved, active-site-directed inhibitors risk simultaneously blocking protective, tumor-suppressive PTPs in healthy tissue, raising the potential for adverse effects.

### 7.2. Future Perspectives

Given SHP2’s clearly context-dependent dual role, future drug discovery should invest as heavily in SHP2 activators as in inhibitors, allowing the pharmacological direction of treatment to be selected based on tumor genetics rather than applying a one-size-fits-all inhibition strategy. Second, moving inhibitor design beyond the catalytic site toward allosteric or other non-active-site regions offers the most promising route to isoform-selective agents that spare tumor-suppressive PTPs in normal tissue. Third, PTP-targeted agents should be further integrated into combination and immunotherapy regimens, building on encouraging early precedent. In RAS- and RTK-driven cancers, pairing SHP2 inhibitors with KRAS G12C inhibitors (sotorasib plus RMC-4630) or with EGFR inhibitors (TNO155 plus nazartinib) has yielded encouraging results. Beyond these, targeting PTPN2 has been shown to boost CAR T-cell persistence and effector function against solid tumors, while inhibiting PTPN22 can potentiate the response to anti-PD-1 checkpoint blockade. Future research should further evaluate combination regimens that integrate RTK-targeted therapies, RAS-pathway inhibitors, and immune checkpoint blockade.

Ultimately, achieving safe, selective PTP-targeting agents would unlock an entirely new class of cancer therapeutics, one capable of addressing numerous malignancies that remain underserved by kinase-focused drug development.

## Figures and Tables

**Figure 1:**
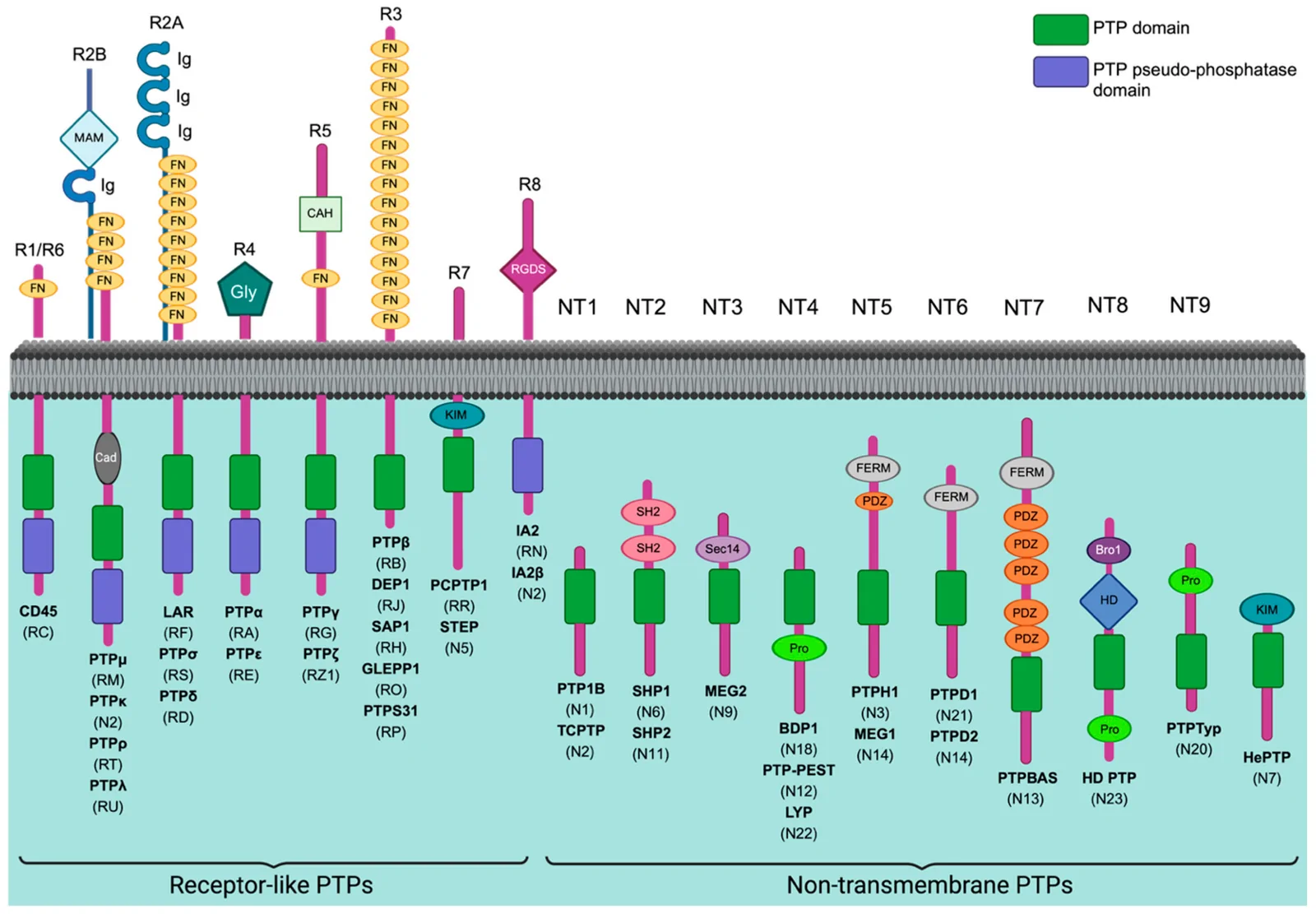
The superfamily of protein phosphatases. Domain architecture of the receptor-like protein tyrosine phosphatases (PTPs; left, R1/R6–R8) and non-transmembrane PTPs (right, NT1–NT9). Each PTP is shown with its catalytic PTP domain (green) and, where present, a non-catalytic PTP pseudo-phosphatase domain (purple), together with accessory extracellular and intracellular domains that mediate ligand binding, membrane anchoring, and protein–protein interactions, including immunoglobulin-like (Ig), fibronectin type III (FN), MAM, carbonic anhydrase-like (CAH), glycine-rich (Gly), RGDS, cadherin-like (Cad), Src homology 2 (SH2), FERM, PDZ, Sec14, kinase-interacting motif (KIM), Bro1, HD, and proline-rich (Pro) domains. Standard gene family designations (R1–R8, N1–N23) are indicated in parentheses beneath each PTP name. [Created in BioRender. Perla, S. (2026) (https://BioRender.com/wao823p, accessed on 13 September 2026) is licensed under CC BY 4.0].

**Figure 2:**
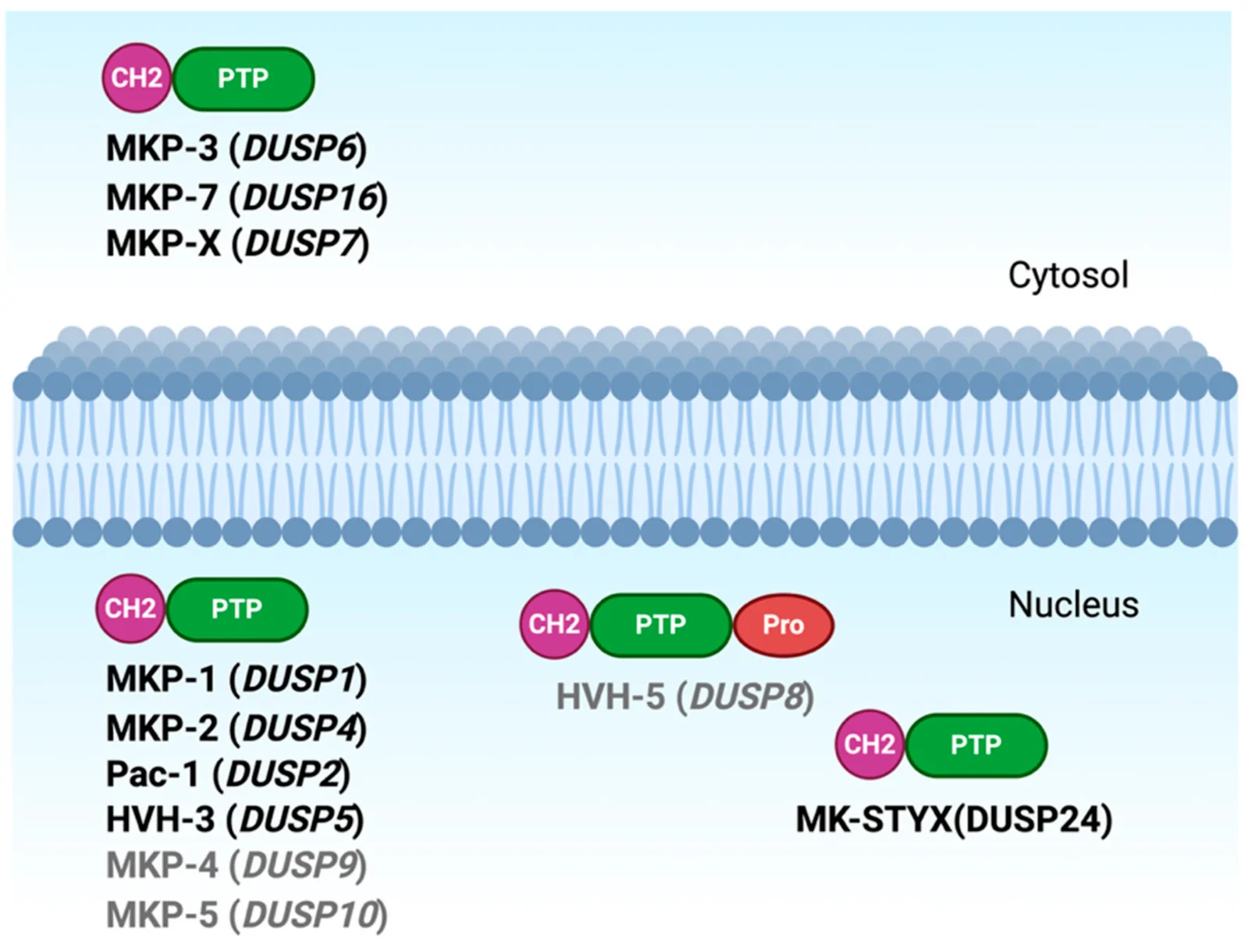
The superfamily of protein phosphatases and dual-specificity phosphatases. Subcellular distribution and domain organization of the dual-specificity phosphatases (DUSPs), also known as mitogen-activated protein kinase phosphatases (MKPs). Cytosolic DUSPs (top; MKP-3/DUSP6, MKP-7/DUSP16, MKP-X/DUSP7) and nuclear DUSPs (bottom; MKP-1/DUSP1, MKP-2/DUSP4, Pac-1/DUSP2, HVH-3/DUSP5, MKP-4/DUSP9, MKP-5/DUSP10, HVH-5/DUSP8, MK-STYX/DUSP24) are shown relative to the plasma membrane, each comprising an N-terminal CH2 (rhodanese-like) domain and a catalytic PTP domain, with a proline-rich (Pro) domain additionally present in HVH-5/DUSP8. Gray text/shading denotes family members not discussed further in this review. [Created in BioRender. Perla, S. (2026) (https://BioRender.com/1mtcpmp) is licensed under CC BY 4.0].

**Figure 3:**
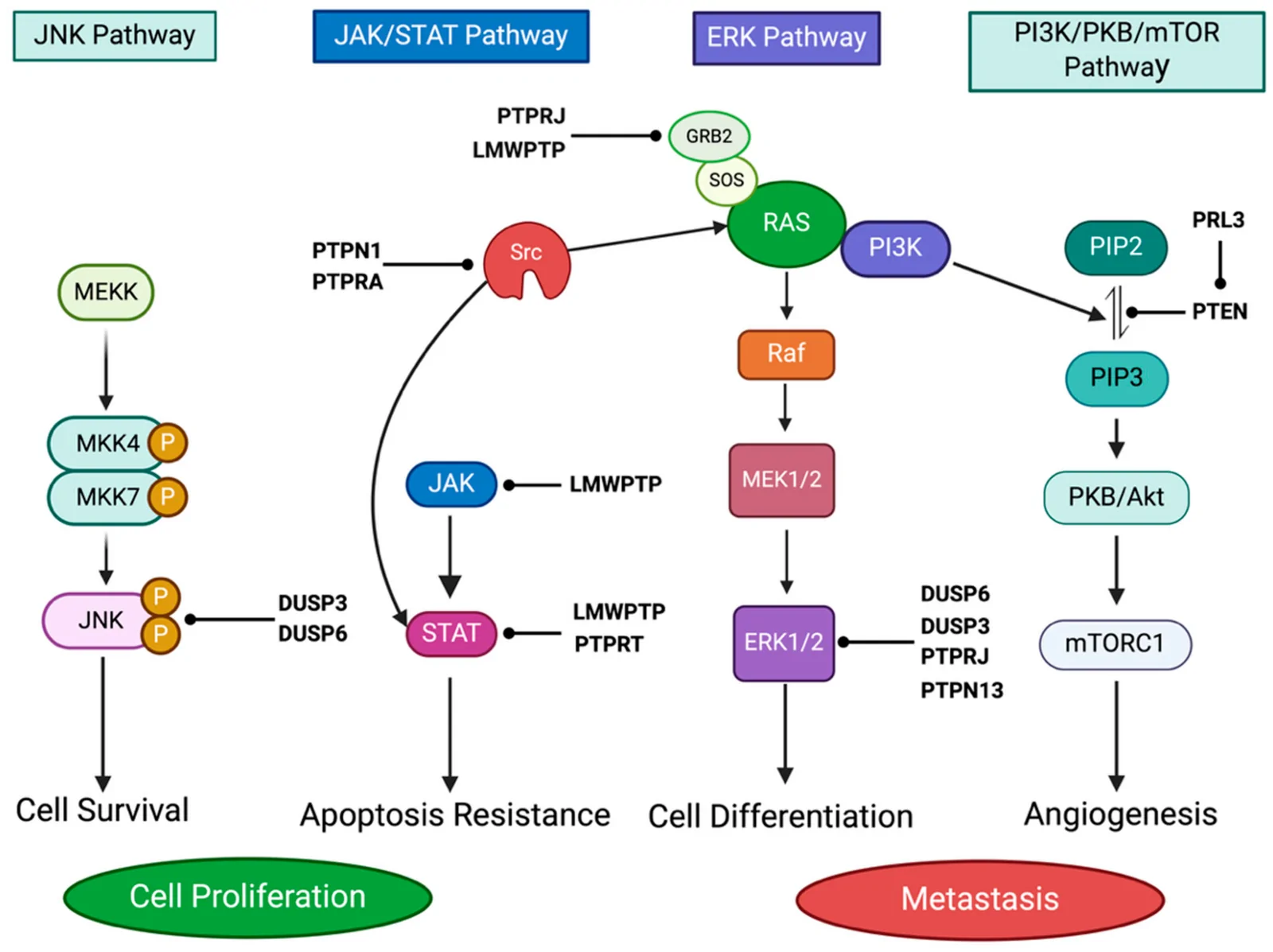
Schematic overview of signaling pathways regulated by PTPs in cancer. PTP effects on proteins are indicated by lines with dots and downstream signaling effects are indicated by lines with arrows. Four major oncogenic signaling cascades are shown: the c-Jun N-terminal kinase (JNK) pathway (MEKK→MKK4/MKK7→JNK, negatively regulated by DUSP3 and DUSP6), the Janus kinase/signal transducer and activator of transcription (JAK/STAT) pathway (Src→JAK→STAT, negatively regulated by PTPN1, PTPRA, low-molecular-weight PTP [LMWPTP], and PTPRT), the extracellular signal-regulated kinase (ERK) pathway (GRB2/SOS→RAS→Raf→MEK1/2→ERK1/2, negatively regulated by PTPRJ, LMWPTP, DUSP3, DUSP6, and PTPN13), and the phosphoinositide 3-kinase/protein kinase B/mechanistic target of rapamycin (PI3K/PKB/mTOR) pathway (PI3K→PIP2/PIP3→PKB/Akt→mTORC1, negatively regulated by PTEN and promoted by PRL3). Together, these pathways converge to drive cell survival, apoptosis resistance, cell proliferation, cell differentiation, angiogenesis, and metastasis. Arrows indicate the direction of downstream signaling, whereas lines ending in dots indicate PTP-mediated regulatory effects. [Created in BioRender. Perla, S. (2026) https://BioRender.com/ou8lw12].

**Figure 4:**
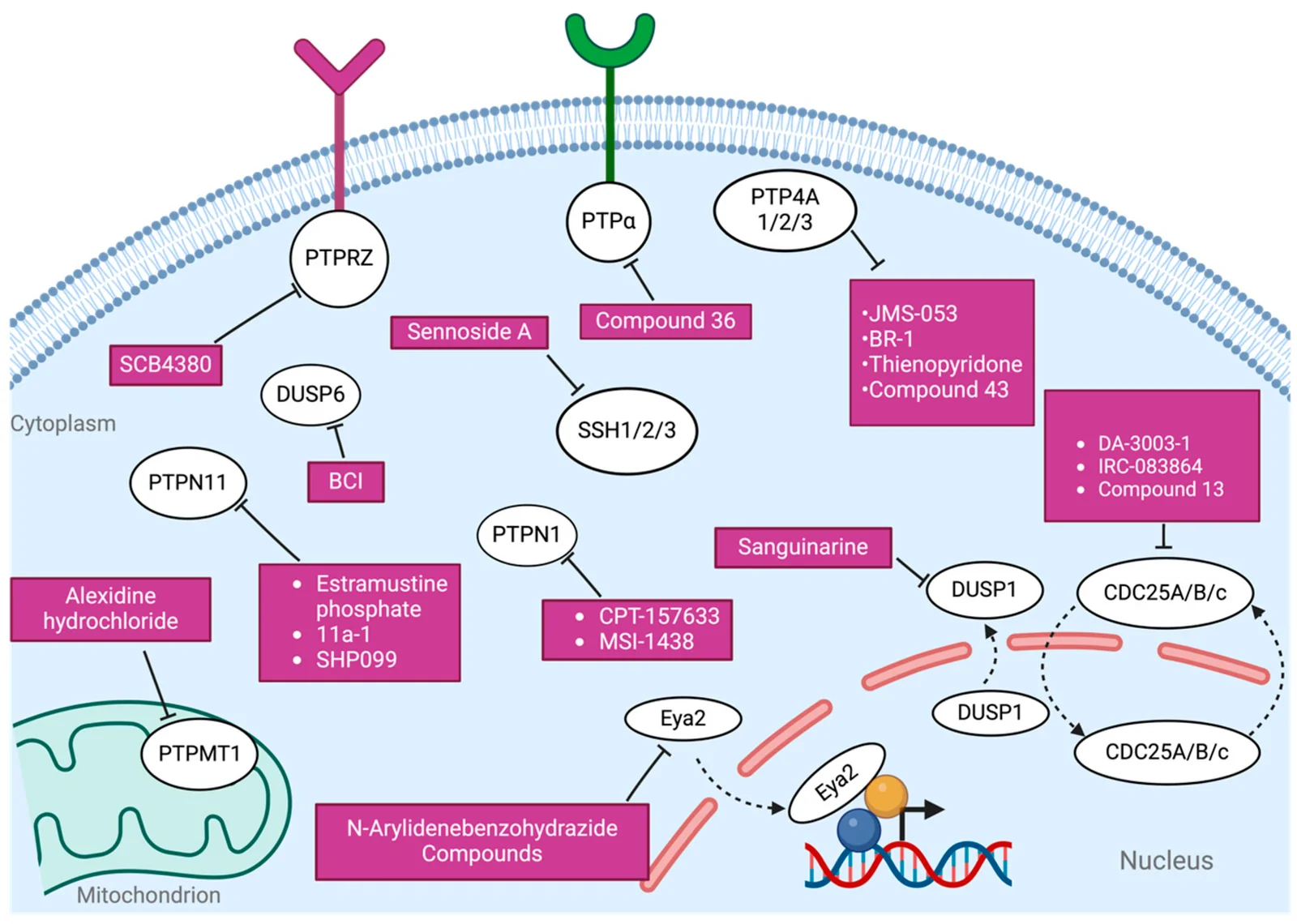
Subcellular localization of PTP inhibitors. A schematic representation of the subcellular localization of PTPs and corresponding SMIs. Selected oncogenic PTPs are shown at their sites of action—the plasma membrane (PTPRZ, PTPα, PTP4A1/2/3, DUSP6), cytoplasm (PTPN11/SHP2, PTPN1/PTP1B, SSH1/2/3), mitochondrion (PTPMT1), and nucleus (Eya2, DUSP1, CDC25A/B/C)—together with representative small-molecule inhibitors (SMIs, magenta boxes) reported to target each phosphatase, including SCB4380 (PTPRZ), Compound 36 (PTPα), JMS-053, BR-1, thienopyridone, and Compound 43 (PTP4A1/2/3), BCI (DUSP6), estramustine phosphate, 11a-1, and SHP099 (PTPN11/SHP2), CPT-157633 and MSI-1436 (PTPN1/PTP1B), sanguinarine (DUSP1), sennoside A (SSH1/2/3), alexidine hydrochloride (PTPMT1), N-arylidenebenzohydrazide compounds (Eya2), and DA-3003-1, IRC-083864, and Compound 13 (CDC25A/B/C). Arrows with flat bars indicate inhibition of the corresponding PTP or downstream effector. All inhibitors are shown in dark magenta boxes. [Created in BioRender. Perla, S. (2026) (https://BioRender.com/t84d072) is licensed under CC BY 4.0].

**Table 1:** List of tumor-suppressor PTPs and cancer.

Cancer	PTP	Signaling Pathway	Ref
Breast	PTPRO	P53/FOXM1	[85]
Ovarian	DUSP4	MAPK	[86]
Leukemia	DUSP6	MAPK	[87]
Breast	PTPN12	EGFR, HER2, mTOR	[88]
NSCLC, Colorectal, Prostate, Lymphoma, Breast, Liver, Ovarian	PTPRK	EGFR, AKT	[89,90,91,92]
Colorectal Cancer	PTPRF	Wnt	[93]
Ovarian, Breast, Leukemia	PTPRG	PI3K/AKT, FGFR, AXL, BCR:ABL1	[93,94,95]
Prostate, Breast	PTEN	PI3K/AKT	[96,97]
Leukemia, Lymphoma	SHP1	AKT-β-catenin, BCR signaling pathway	[98,99]

## Data Availability

No new data were created or analyzed in this study. Data sharing is not applicable to this article.

## Abbreviations

The following abbreviations are used in this manuscript:

AML	Acute myeloid leukemia
CAR	Chimeric antigen receptor
DUSP	Dual-specificity phosphatases
DUSP1	Dual-specificity phosphatase 1
DUSP16	Dual-specificity phosphatase 16
DUSP2	Dual-specificity phosphatase 2
DUSP6	Dual-specificity phosphatase 6
EMT	Epithelial–mesenchymal transition
HCC	Hepatocellular carcinoma
MKP	Mitogen-activated protein kinase phosphatase
NLS	Nuclear localization signal
NSCLC	Non-small cell lung cancer
PPM	Mg^2+^/Mn^2+^-dependent protein phosphatase family
PPP	Phosphoprotein phosphatase family
PROTAC	Proteolysis-targeting chimera
PTP1B	Protein tyrosine phosphatase 1B
SH2	Src homology region 2
SHP2	SH2-containing protein tyrosine phosphatase 2
TAMs	Tumor-associated macrophages
TC-PTP	T-cell protein tyrosine phosphatase
YAP	Yes-associated protein
